# Next-Generation Sequencing of Ancient and Recent Fungarium Specimens

**DOI:** 10.3390/jof8090932

**Published:** 2022-09-02

**Authors:** Andrew N. Miller, Jason Karakehian, Daniel B. Raudabaugh

**Affiliations:** 1 Illinois Natural History Survey, University of Illinois at Urbana-Champaign, 1816 South Oak Street, Champaign, IL 61820, USA; 2 Department of Plant Biology, University of Illinois at Urbana-Champaign, 505 South Avenue, Urbana, IL 61801, USA; 3 Department of Botany and Plant Pathology, Purdue University, 915 West State Street, West Lafayette, IN 47907, USA

**Keywords:** DNA barcode, fungi, internal transcribed spacer, Sanger sequencing, taxonomy, type specimens

## Abstract

Fungaria are an unmatched resource for providing genetic data from authoritative, taxonomically-correct fungal species, especially type specimens. These specimens serve to anchor species hypotheses by enabling the correct taxonomic placement of taxa in systematic studies. The DNA from ancient specimens older than 30 years is commonly fragmented, and sometimes highly contaminated by exogenous, non-target fungal DNA, making conventional PCR amplification and Sanger sequencing difficult or impossible. Here, we present the results of DNA extraction, PCR amplification of the ITS2 region, and Illumina MiSeq Nano sequencing of nine recent and 11 ancient specimens, including seven type specimens. The taxa sampled included a range of large and fleshy, to small and tough, or small, melanized specimens of *Discina*, *Gyromitra*, *Propolis*, *Stictis*, and *Xerotrema*, with a culture of *Lasiosphaeria* serving as a positive control. DNA was highly fragmented and in very low quantity for most samples, resulting in inconclusive or incorrect results for all but five samples. Taxonomically-correct sequences were generated from the holotype specimens of *G. arctica*, *G. korshinskii*, and *G. leucoxantha*, from the neotype of *G. ussuriensis*, and from the positive control. Taxonomic assignments were confirmed through morphology, top BLASTn hits, and maximum likelihood phylogenetic analyses. Though this study was not cost-effective due to the small number of samples submitted and few generating correct sequences, it did produce short DNA barcode fragments for four type specimens that are essential for their correct taxonomic placement in our ongoing systematic studies.

## 1. Introduction

Fungarium specimens offer an unprecedented diversity in the taxonomic breadth and depth of taxa that cannot be compared to or achieved through even the largest and broadest fieldwork studies aimed at obtaining fresh specimens, mainly due to the ephemeral nature of fungi. Fungaria are an untapped resource that contain the most precious and valuable specimens through their holdings of type specimens [1], which represent the only absolute link between a fungal species and its taxonomic name. However, mining genetic data from type specimens for DNA barcoding and genomic studies is often difficult due to the age, preservation, and storage of these often ancient (>30 years old) specimens [2]. Over time, DNA from century-old specimens becomes highly degraded into fragments that are generally less than 500 bp [3,4,5]. This fragmentation can be accelerated by the use of a variety of chemical pesticides that were often employed in fungaria to reduce the damage to the specimens by insects [6,7]. Another constraint is that the amount of physical material associated with type specimens is often limited, so some fungaria are restrictive on whether they allow destructive sampling for molecular studies. Lastly, specimens have historically been stored in compact conditions due to space limitations, and under environmental conditions to preserve their morphological features, not to optimize DNA quantity or quality [6,8]. This has led to intact exogenous DNA by other fungi in the form of conidia, spores, and hyphae contaminating ancient specimens. Universal primers used in DNA barcoding do not discriminate between target and contaminate DNA in the amplification process, and next-generation sequencing is very sensitive and highly impartial when generating its final products.

Attempts to DNA barcode large numbers of recent (<30 years old) fungarium specimens using Sanger sequencing have been relatively successful [9,10,11]. Next-generation sequencing is commonly employed for estimating fungal diversity from environmental samples, and for whole genome sequencing of recent and ancient fungarium specimens [12]. However, it has not been widely used for obtaining molecular barcode data from fungarium specimens, especially ancient type specimens that are potentially contaminated by other fungi, and in which the DNA is usually fragmented, making PCR amplification and traditional Sanger sequencing difficult or impossible [8]. Only a few other studies to date have attempted to use next-generation sequencing to obtain DNA barcode data from fungarium specimens [8,11,13,14]. Forin et al. [8] were moderately successful in using Illumina MiSeq technology to sequence the ITS2 region from ancient *Peziza* specimens, and Forin et al. [13] successfully sequenced the ITS1 and/or ITS2 regions from ancient *Rosellinia* specimens held in Saccardo’s mycological herbarium. Olds et al. [11] were highly successful in using the Illumina platform to sequence the ITS2 region from more recent specimens, mainly from the Denver Botanical Garden, but only moderately successful when sequencing ancient specimens collected between 1910–1990. Runnel et al. [14] were successful in using PacBio sequencing to generate long-read ITS-LSU sequences from recent specimens collected between 2015–2020.

The goal of this study was to generate ITS2 sequence data from recalcitrant ancient and recent dried voucher specimens, especially type material, employing Illumina next-generation sequencing technology. Providing DNA sequence data for authenticated voucher specimens connects the morphological descriptions with the molecular characters, which is vitally important for affixing taxonomic names in systematic studies. These short DNA barcode fragments are essential for the correct taxonomic placement of type specimens in our ongoing systematic studies, and provide a valuable confirmation of distributional patterns in fungi.

## 2. Materials and Methods

### 2.1. Taxa Selection

The taxa included in this study were selected based on one or more of the following conditions: (1) inclusion of both recent and ancient specimens; (2) sampling a range of large and fleshy, to small and tough, or small, highly melanized specimens; (3) the presumption that standard PCR amplification would fail based on ancient specimens; (4) documented previous PCR amplification failure regardless of age; and (5) the importance of obtaining even a small fragment of the DNA sequence for type specimens. As such, one specimen of *Propolis*, one specimen of *Discina*, three specimens of *Stictis*, six specimens of *Gyromitra*, and eight specimens of *Xerotrema* were selected, along with a culture of *Lasiosphaeria lanuginosa*, which served as the positive control (Table 1). Morphological analyses of the ascocarps, asci, and ascospores were conducted to confirm taxonomic identification for all specimens, except for five (*G. arctica*, *G. korshinskii*, *G. leucoxantha*, *G. perlata*, *G. ussuriensis* (LE 179636)) in which only enough material for DNA extraction was sent. Regardless, *G. arctica*, *G. korshinskii*, and *G. leucoxantha* represent type specimens, making confirmation of the taxonomic assignment based on morphology irrelevant. A morphological examination of *G. perlata* has already been thoroughly documented [15]. A morphological analysis of *G. ussuriensis* (LE 179636) would be pointless, since it is an immature specimen consisting of only a few pieces of a single, highly fragmented ascoma (Eugene Popov pers. comm.).

### 2.2. DNA Extraction, Quantification, and Fragmentation Analysis

Small samples (10–100 mg) of fungal tissue from dried voucher specimens (or a culture in the case of the positive control) were placed in 1.5 µL centrifuge tubes either by one of the authors or by the curator/collections manager at the lending institution. One of the following (or both for one sample) DNA extraction methods was used: an EZNA^®^ Microelute Genomic DNA Kit (Omega Bio-tek), or modification of the NaOH extraction [16] (Table 2). Fungal tissue was repeatedly frozen at −20 °C and thawed at room temperature at least three times in either lysis buffer or 50–200 µL of 0.5 M NaOH to help break up fungal cell walls, and was then ground using a micropestle and a tissue grinder. For the EZNA kit, extraction followed the manufacturer’s instructions, except DNA was diluted in only 30 µL of DNA-free water in the final step. For the NaOH method, ground extract was incubated at 4 °C for 30 min, centrifuged at 16,800× *g* for 2 min, and 5 µL of the resulting supernatant was added to 45 µL of 100 mM Tris-HCl buffered with NaOH to pH 8.5–8.9. In one sample (CUP-A-030055), this 50 µL DNA solution was extracted a second time using the EZNA kit. The DNA for some samples (S-F-11771; UPS-F-144599; and all *Propolis*, *Stictis*, and *Xerotrema*) was further cleaned and concentrated using a Zymo DNA Clean & Concentrator^TM^ kit (Zymo Research) (Table 2). All procedures were conducted under standard conditions on a 10% bleach-disinfected laboratory benchtop using UV-sterilized materials. Final DNA extracts were subjected to quantification using a Qubit fluorometer (Thermo Fisher Scientific, Waltham, MA, USA), and DNA fragmentation was assessed on a 5300 high-sensitivity Fragment Analyzer (Advanced Analytical Technologies, Inc., Santa Clara, CA, USA).

### 2.3. PCR Amplification and Sanger Sequencing

PCR amplification for eventual Sanger sequencing was completed in 25 µL total reaction volumes consisting of the following: 12.5 μL GoTaq^®^ Green Master Mix (Promega); 2.5 µL bovine serum albumin (New England Biolabs); 2.5 µL of 50% dimethyl sulfoxide (Fisher Scientific); 1.5 μL of each 10 μM primer combination, ITS1F-ITS2 or ITS3-ITS4 [17,18]; and 5 μL of DNA. PCR was completed on a Bio-Rad C1000 thermal cycler under the following conditions: initial denaturation at 94 °C for 2 min, followed by 40 cycles of 94 °C for 30 s, 41 °C for 20 s, 72 °C for 1 min, with a final extension step of 72 °C for 10 min. Gel electrophoresis on a 1% TBE agarose gel stained with ethidium bromide was used to verify the presence of a PCR product. Purification was completed using a Wizard^®^ SV Gel and PCR Clean-Up System (Promega) with the purified product diluted in 20–30 µL DNA-free water. A BigDye^®^ Terminator 3.1 cycle sequencing kit (Applied Biosystems Inc., Waltham, MA, USA) was used to sequence the separate ITS1 and ITS2 regions in both directions using the above PCR primers on an Applied Biosystems 3730XL high-throughput capillary sequencer at the Roy J. Carver Biotechnology Center at the University of Illinois.

### 2.4. PCR Amplification and Next-Generation Sequencing

Comparisons between the ITS1 and ITS2 regions have shown the phylogenetic signal between these markers to be similar [19,20], but the ITS2 is often preferred, given the greater number of reference sequences available and the potential for introns in the ITS1 region [21,22]. Although the ITS1 region is more variable and better at resolving species in *Gyromitra* [23], it also contains large introns in some groups in this genus, making it very difficult to PCR-amplify ancient specimens with fragmented DNA (Miller unpub. data).

Purified DNA samples were analyzed using a high-sensitivity gel on a 5300 Fragment Analyzer to visualize the degree of DNA fragmentation. The ITS2 region was amplified using Fluidigm reagents and barcodes in a two-step PCR procedure. In the first step, the ITS2 region was amplified for 35 cycles using the fITS7 plus CS1 Fluidigm primer pad and the ITS4 plus CS2 Fluidigm primer pad [18,24]. The second step consisted of 14 cycles attaching the 10 bp sample specific identification barcode and Illumina adaptor barcodes. The final amplicon construct consisted of the following: Illumina i5 adaptor—CS1—fITS7 primer—region of interest—ITS4 primer—CS2—sample specific 10 bp barcode—Illumina i7 adaptor. The 5300 Fragment Analyzer was used to visualize the amplicons after each PCR step to confirm the presence of amplicons. The final amplicons were sequenced on Illumina MiSeq v3 platform rapid 2 × 250 nt PE NANO v2 paired-end reads (San Diego, CA, USA). All PCR amplification and sequencing steps were performed at the Roy J. Carver Biotechnology Center at the University of Illinois.

### 2.5. Next-Generation Sequencing Data Processing, OTU Identification, and Taxonomic Assignment

The resulting Illumina sequences were processed with DADA2 [25] using both forward and reverse amplicons. The *filterAndTrim* function was set up as follows: maxN = 0, maxEE = c(2,2), truncQ = 1, and rm.phix = True. The *LearnErrors* function was completed on both forward and reverse reads. After running the core *dada* function, forward and reverse reads were merged with the *mergePairs* function. Chimeras were removed using the *removeBimeraDenovo* function with the method set to *consensus*. All steps were completed with R version 4.1.2 [26]. A FASTA file of the *dada2* amplicon sequence variants (ASVs) was generated using SeaView [27]. The FASTA file was compared against the NCBI nucleotide database, excluding environmental sequences, using NCBI BLASTn [28] to determine the sequence identity. The NCBI XML file/s were downloaded and parsed using the NCBI BLAST parser tool [29], and the best NCBI BLASTn match was retained. In addition to ASVs (99% sequence similarity), we also examined the results via Organized Taxonomic Units (OTUs) at 97% sequence similarity using DECIPHER [30] in R statistical software. In short, an initial sequence alignment was completed with the function, *AlignSeqs*; a distance matrix was computed from the aligned sequences using the function, *DistanceMatrix*; and 97% OTU clustering was completed using the function, *IdClusters*, with method set to *complete* and the cutoff set to *0.03*. Raw Illumina reads have been deposited into the NCBI Sequence Read Archive (SRA) database (BioProject ID: PRJNA870474, http://www.ncbi.nlm.nih.gov/bioproject/870474).

### 2.6. Phylogenetic Analyses

Verification of the taxonomic name assigned to each specimen can only be completed if well-annotated, taxonomically-accurate sequences for that taxon occur in GenBank. Since only four of our 12 sampled taxa have available sequences in GenBank (Table 2), it was necessary to conduct phylogenetic analyses to verify the taxonomic assignment suggested by morphology and BLASTn sequence similarity. Alignments of the ITS2 region (or the entire ITS for *G. korshinskii*) were assembled using MUSCLE^®^, as implemented in Sequencher 5.4.6 (Gene Codes Corporation, Ann Arbor, MI, USA) for each target species and its most closely-related taxa based on BLASTn results and previous phylogenetic analyses ([31,32] Miller unpub. data). The best-fit model of evolution was determined to be the general time reversible (GTR) model [33] by jModeltest [34,35] based on the Akaike information criterion (AIC) [36]. A maximum likelihood (ML) analysis with 1000 bootstrap replicates was performed using PhyML, as implemented in Seaview 5.0.5 [37], with all parameters optimized and the GTR model. Clades with bootstrap values (BV) ≥70% were considered significant and strongly supported [38].

## 3. Results and Discussion

### 3.1. Taxa Sampled

A range of recent-to-ancient specimens that varied from large and fleshy (i.e., *Discina*, *Gyromitra*), to small and tough (i.e., *Propolis*, *Stictis*), or minute and melanized (i.e., *Xerotrema*), were selected for this study. Specimens were collected between 1863–2009, except for the positive control culture isolated in 2021, and, therefore, ranged in age from 13–159 years (Table 1). It was presumed that all specimens have been stored under typical fungarium conditions of moderate temperature and low humidity at the 11 institutions from which the specimens were loaned. No obvious signs of additional fungal growth contaminating the target specimens was observed on the outside of the sampled tissue. The taxonomic names listed in Table 1 were confirmed through morphological examination when possible. *Gyromitra ussuriensis* (LE 179636) consists of a highly fragmented specimen that is immature (Eugene Popov, pers. comm.), so it could only be identified through molecular data.

### 3.2. DNA Extraction, Quantification, and Fragmentation

**Figure 1:**
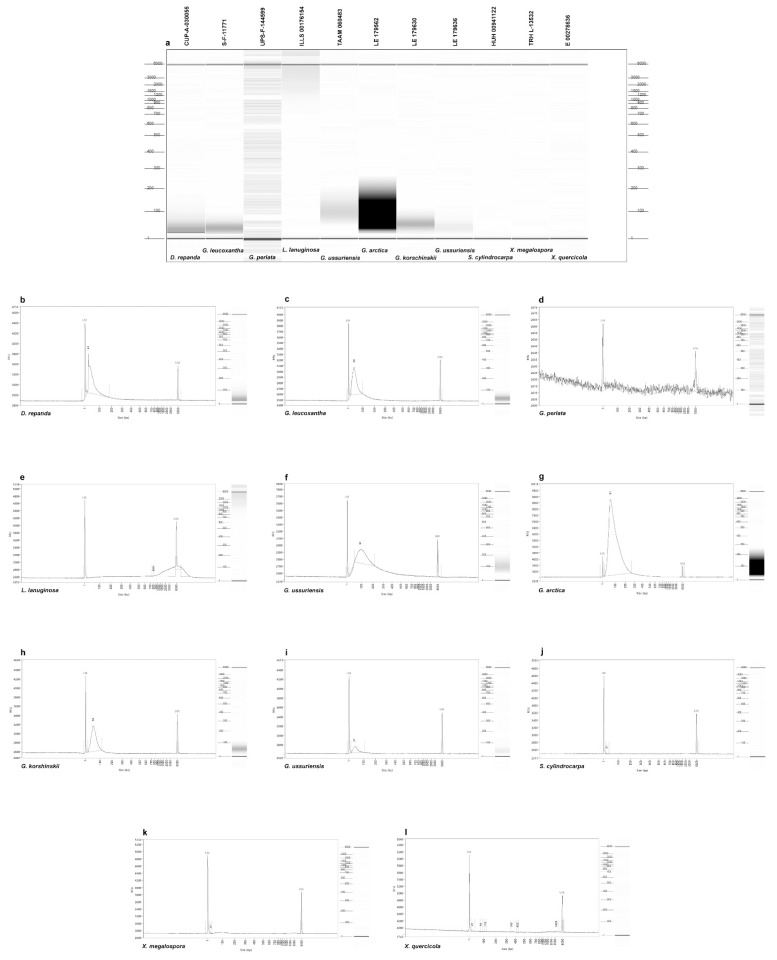
Gel image and graphs showing DNA fragmentation of 11 samples from fragment analyzer. Scales on left and rights sides of gel image indicate base pairs; scale on left side of charts indicates amount of fragmentation in relative fluorescence units; size of fragments is shown in base pairs along bottom; gel image for each sample is repeated to the right of chart with scale in base pairs. (**a**) Gel image showing DNA fragmentation of 11 samples. (**b**) *Discina repanda*. (**c**) *Gyromitra leucoxantha*. (**d**) *Gyromitra perlata*. (**e**) *Lasiosphaeria lanuginosa*. (**f**) *Gyromitra ussuriensis*. (**g**) *Gyromitra arctica*. (**h**) *Gyromitra korshinskii*. (**i**) *Gyromitra ussuriensis*. (**j**) *Sticta cylindrocarpa*. (**k**) *Xerotrema megalospora*. (**l**) *Xerotrema quericola*.

Extraction using either the EZNA kit, which results in lower amounts of higher-quality DNA, or the NaOH method, which results in higher amounts of lower-quality DNA, did not seem to affect the overall quantity of the final DNA, although the sample size was very limited (Table 2). Cleaning and concentrating the DNA after extraction with a Zymo kit did not increase the success of PCR amplification and sequencing. Quantification using Qubit was too low to detect for most samples, but ranged from 1.65–6.56 ng/µL for the four samples with detectable DNA. Fragment analyses were run for 11 samples (Figure 1a–l), and all were fragmented except for the control, which showed no fragmentation between 20–800 bp (Figure 1e). DNA was undetectable and highly fragmented with no discernible peaks between 20–6000 bp, and the resulting sequences were inconclusive for specimens of *S. fulva*, *X. megalospora*, and *X. quericola* (Figure 1j–l). Although *G. perlata* had the highest amount of DNA, it also was the most fragmented, explaining why PCR amplification failed for this sample (Figure 1d). Fries’ material showed no signs of ever being treated with chemicals (Åsa Kruys pers. comm.), so age is the most likely explanation for its high rate of DNA fragmentation. The remaining *Discina* and five *Gyromitra* specimens had fragmented DNA, but obvious peaks between 20–300 bp, allowing the generation of successful PCR products and taxonomically correct ITS2 sequences for all five *Gyromitra* specimens (Figure 1b,c,f–i).

### 3.3. Sanger Sequencing

Although PCR amplification was attempted for all samples, only *G. arctica*, *G. korshinskii*, and the control successfully PCR-amplified and produced subsequent Sanger sequences in both directions. Whereas the entire ITS region was PCR-amplified in two pieces and sequenced for *G. korshinskii* and the control, only the ITS2 region was successfully PCR-amplified and was sequenced for *G. arctica* (Table 2). These three consensus Sanger sequences, which contain accompanying chromatograms, had zero or one nucleotide difference between them and their Illumina-generated sequences, which lack chromatograms. Sanger sequences provide additional data to support our taxonomic assignments, and serve as a proof-of-concept for the Illumina sequencing.

### 3.4. Next-Generation Sequencing

**Figure 2:**
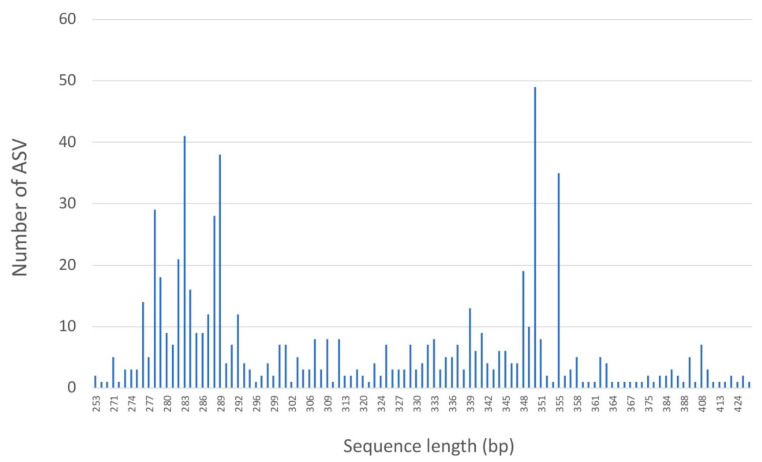
Distribution of ITS2 sequence lengths across all samples.

PCR amplification was successful for all samples except *G. perlata*, which happens to be the oldest specimen, from around 1863 (Table 1). The Illumina MiSeq Nano sequencing generated a total of 891,996 sequences: 445,998 in the forward direction and 445,998 in the reverse, of which, 77% of the sequences passed quality filtering. In total, there were 312,683 contig sequences, with an average of 16,457 contig sequences per sample (Table 2). Prior to removing chimeras, the unique contig ASVs per sample ranged from 6 to 93 per sample. In total, 88 bimeras were detected, resulting in 609 unique ASVs across all samples. These ASVs were clustered into 283 OTUs at 97% similarity. After OTU clustering, it was noted that every OTU composed of multiple ASVs contained consistent final taxonomic determinations. The final sequence length for the ITS2 region ranged from 253 bp to 446 bp (Figure 2).

### 3.5. Contamination

**Figure 3:**
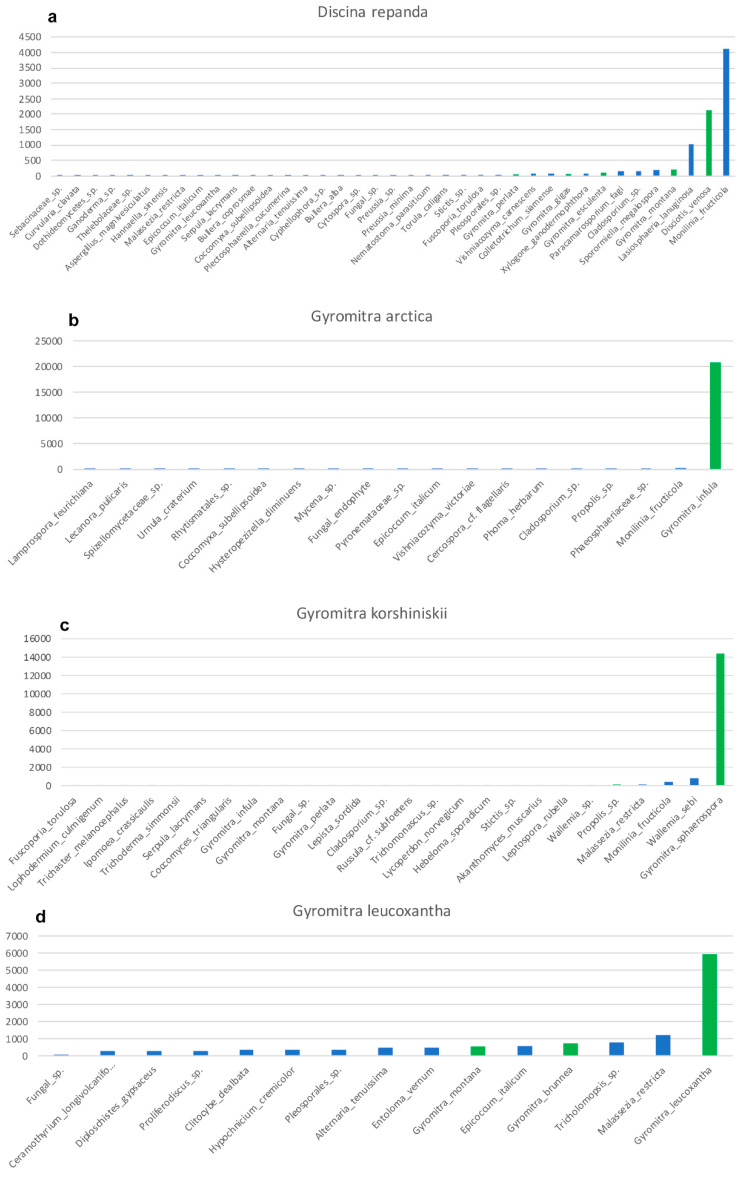

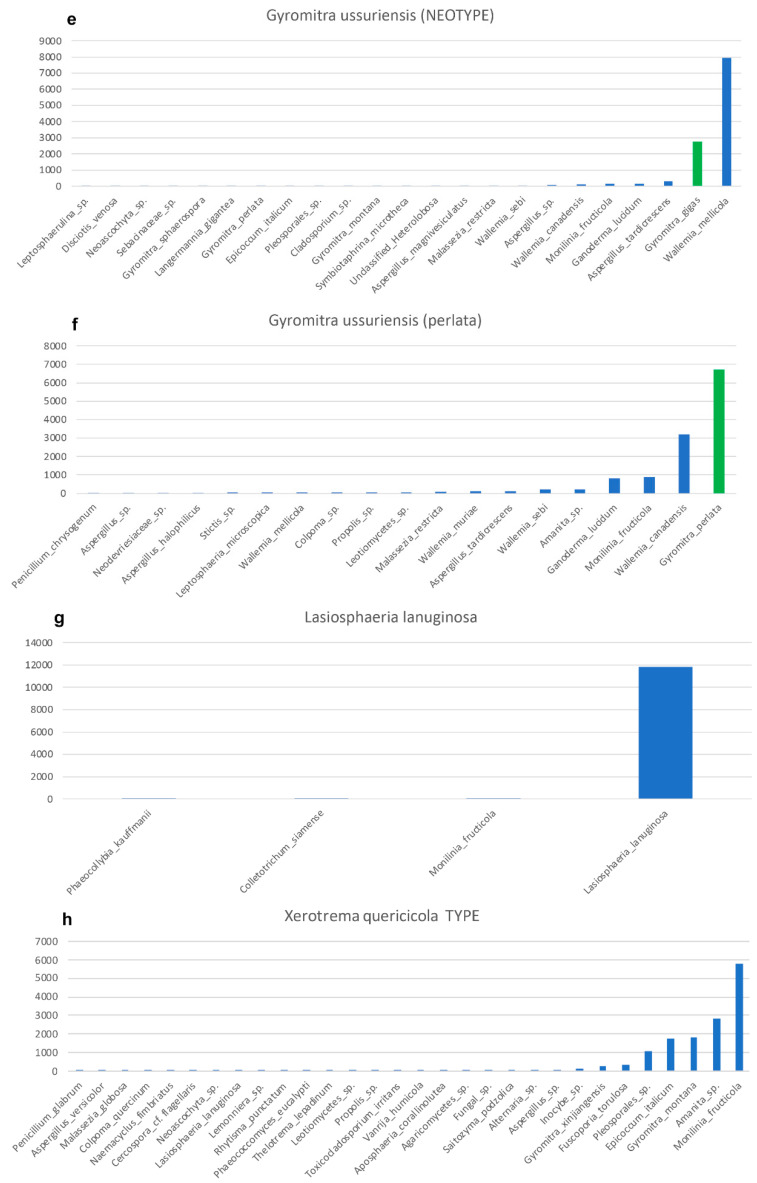
Taxonomic assignments shown as increasing number of Illumina-generated ITS2 sequences for each sample. Bars are shown in green for all species in the genus, *Gyromitra*. (**a**) *Discina repanda*. (**b**) *Gyromitra arctica*. (**c**) *Gyromitra korshinskii*. (**d**) *Gyromitra leucoxantha*. (**e**) *Gyromitra ussuriensis*. (**f**) *Gyromitra ussuriensis*. (**g**) *Lasiosphaeria lanuginosa*. (**h**) *Xerotrema quericola*.

Ancient, and even sometimes recent, specimens can be heavily contaminated with exogenous fungal material, including conidia, spores, and hyphae. These fungal contaminates are PCR-amplified and sequenced, typically in low abundance, during Illumina sequencing. All of our samples contained sequences from contaminate fungi (Figure 3), including *Wallemia* spp., which are highly xerotolerant or xerophilic basidiomycetes that commonly occur in osmotically-challenged environments, as would be found in modern fungaria [39]. The fact that 67% of the sequences from *Gyromitra ussuriensis* were *Wallemia mellicola* (Figure 3e) suggests better PCR amplification, possibly due to less DNA fragmentation of the contaminate or a high amount of contaminate occurring on the specimen compared to the target. However, since 23% of the sequences were the target fungus (Table 2), with the third highest level of sequences at 2.5% (Figure 3e), we feel confident making this taxonomic assignment. All samples, except *G. leucoxantha*, contained *Monilia fructicola*, which is not surprising, since our lab was working with cultures of this peach pathogen at the time we conducted this study (Figure 3). We were also sequencing isolates of *Collectotrichum siamense*, a pathogen of apples, which also shows up in the control, although at a very low abundance (Figure 3g). Sequences of target species, albeit it in a very low abundance, occurred in two other unrelated samples (Figure 3c,e), indicating either the possibility of minor cross-over occurred during our molecular procedures, or cross-contamination of spores in the fungarium.

### 3.6. Phylogenetic Analyses

*Gyromitra* *arctica*

**Figure 4:**
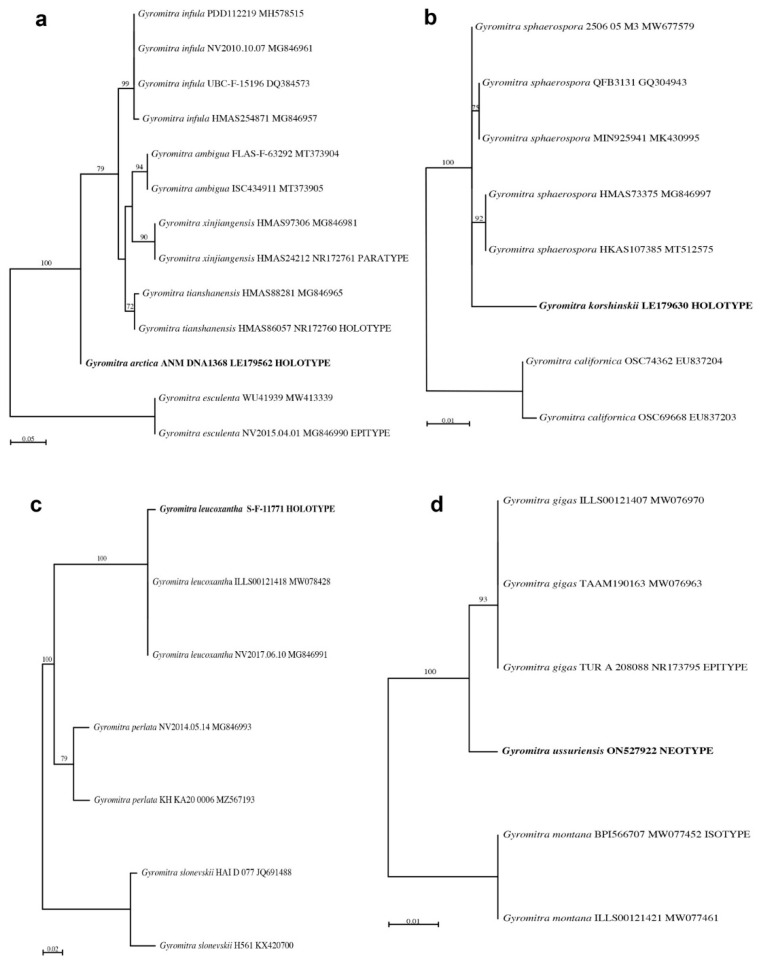
Phylogenetic trees showing the taxonomic placement of the target species based on maximum likelihood analyses of Illumina-generated ITS2 (or ITS in (**b**)) sequences. The taxonomic name followed by the fungarium number and GenBank accession number are given for each taxon. The target species are shown in bold font. Bootstrap values > 50% are shown above branches. (**a**) *Gyromitra arctica*. (**b**) *Gyromitra korshinskii*. (**c**) *Gyromitra leucoxantha*. (**d**) *Gyromitra ussuriensis*.

*Gyromitra arctica* produced sufficient DNA after an EZNA extraction, resulting in a DNA concentration of 3.08 ng/µL based on Qubit analysis. Sanger sequences of the ITS2 region in both directions were obtained, and these had zero bp differences compared to the Illumina sequences. The target species represented 97% of the Illumina sequences (Table 2). Since no sequences of *G. arctica* are available in GenBank, this species BLASTed to *G. infula* (Table 2), which occurs as a closely-related species in our ML analysis (Figure 4a). Although some authors have placed *G. arctica* as a synonym under *G. ambigua* [40,41,42], Species Fungorum and MycoBank recognize it as a separate species, which agrees with our analyses.

*Gyromitra* *korshinskii*

Although the EZNA DNA extraction of *G. korshinskii* resulted in a low amount of DNA, PCR amplification and subsequent Sanger and Illumina sequencing produced sequences that did not differ from one another. The target species represented 87% of the Illumina sequences (Table 2). Since no sequences of *G. korshinskii* are available in GenBank, this species BLASTed to *G. sphaerospora* (Table 2), which occurs in a strongly-supported clade with *G. korshinskii* in our ML analysis (Figure 4b). Although *G. korshinskii* has been placed as a synonym under *G. sphaerospora* [43], Species Fungorum and MycoBank recognize it as a separate species, and additional studies are needed to determine if *G. korshinskii* should be recognized as a distinct species.

*Gyromitra* *leucoxantha*

An NaOH DNA extraction followed by a Zymo clean and concentrate of *G. leucoxantha* resulted in a low amount of DNA, but PCR amplification and Illumina sequencing were still successful, with 46% of the sequences representing the target organism (Table 2). Other sequences of *G. leucoxantha* are available in GenBank, and our holotype sequence occurred in a highly-supported clade with these taxonomically-correct sequences (Figure 4c).

*Gyromitra* *ussuriensis*

*Gyromitra ussuriensis* produced sufficient DNA after an EZNA extraction, resulting in a DNA concentration of 2.02 ng/µL based on Qubit analysis. Although this specimen was heavily contaminated with *Wallemia mellicola*, 23% of the sequences were the target fungus (Table 2, Figure 3e). No sequences of *G. ussuriensis* are currently available in GenBank. It was thought to be a synonym of *G. gigas* [44,45,46], but occurs as a distinct species in these analyses (Figure 4d). Since the holotype specimen at VLA of *G. ussuriensis* is missing and presumably lost, and no original material or illustration exists (Eugenis Bulakh, pers. comm.), a neotype has been designated [47].

### 3.7. Costs

The average cost in our lab for the generation of an ITS2 sequence that is Sanger sequenced in both directions is US$6, which includes the in-house costs associated with DNA extraction, PCR amplification, PCR clean-up, generation of fluorescently-labeled sequences and ETOH clean-up of Big Dyes, and the cost for Sanger sequencing on an ABI 3730 at the University of Illinois. This is compared to the per-sample costs at our sequencing center associated with a 2-step PCR amplification ($20), quality check ($4), and the Illumina MiSeq Nano sequencing ($37), or $61 per sample; fragment analysis was an additional $32 per sample. Obviously, more than 20 samples could have been Illumina sequenced, and the 2-step PCR amplification could have been performed in our lab to reduce costs. However, even at ~10X the costs of Sanger sequencing, the ability to obtain an ITS2 sequence from a type specimen is worth the cost for our studies. In the case of *G. leucoxantha* and *G. ussuriensis*, sequences from type specimens were only obtained through Illumina sequencing, justifying the higher cost.

### 3.8. Comparisons with Other Studies

This is one of the first studies to definitively show that Illumina sequencing can provide taxonomically-correct sequences for ancient, highly-fragmented, contaminated type specimens. Though Forin et al. [8] successfully sequenced 23 of 36 ancient *Peziza* specimens from the Saccardo Mycological Herbarium, no type specimens were included in their study. However, Forin et al. [13] successfully sequenced five of nine type specimens of *Rosellinia* from the same herbarium. Both studies used the CTAB method for DNA extraction, which may explain their greater success in sequencing smaller, melanized specimens, versus the NaOH or EZNA kit methods utilized in this study. Though Old et al. [11] did not attempt to sequence any type specimens, their success in obtaining ITS2 sequences from 766 specimens was much greater with recent specimens from 1991–2020 vs. ancient specimens from 1910–1990. Runnel et al. [14] did not include any type specimens in their study of 423 specimens, but showed that PacBio high-throughput sequencing was more successful than Sanger sequencing at a comparable cost.

## 4. Conclusions

Our study attempted to sequence the ITS2 region from recent and ancient DNA from both large and fleshy, and small and tough specimens. Unfortunately, DNA was highly-fragmented in the ancient, smaller specimen of *S. fulva*, and the recent, smaller specimens of *X. megalospora* and *X. quericola*, resulting in inconclusive sequences. However, DNA from the ancient, larger specimens of *Gyromitra* was less fragmented, and we were successful in obtaining taxonomically-correct sequences from four type specimens. The cost of generating ITS2 sequences for only four type specimens in this study was justified by the value we believe these bring for the correct taxonomic placement of names in our systematic studies.

## Figures and Tables

**Table 1:** Samples used in this study with taxonomic name, fungarium number, and year the specimen was collected.

Taxonomic Name	Fungarium Number	Year
*Discina repanda* (Wahlenb.) Sacc.	CUP-A-030055	1898
*Gyromitra arctica* Vassilkov (HOLOTYPE)	LE 179562	1960
*Gyromitra korshinskii* Jacz. (HOLOTYPE)	LE 179630	1886
*Gyromitra leucoxantha* (Bres.) Harmaja (HOLOTYPE)	S-F-11771	1880s?
*Gyromitra perlata* (Fr.) Harmaja (NEOTYPE)	UPS-F-144599	1863?
*Gyromitra ussuriensis* Lj.N. Vassiljeva (NEOTYPE)	TAAM 060483	1961
*Gyromitra ussuriensis*	LE 179636	1960s?
*Lasiosphaeria lanuginosa* (P. Crouan and H. Crouan) A.N. Mill. and Huhndorf (+control)	ILLS 00176154	2021
*Propolis* sp.	HUH 00941116	1974
*Stictis cylindrocarpa* Peck	ILLS 00169337	1935
*Stictis fulva* Peck (ISOTYPE)	HUH 00941122	1879
*Stictis fulva*	HUH 00941117	1897
*Xerotrema megalospora* Sherwood and Coppins	TRH L-13532	2009
*Xerotrema megalospora*	UBC L-63079	2005
*Xerotrema megalospora*	ASU L572580	1993
*Xerotrema megalospora*	E 00948846	1999
*Xerotrema megalospora*	E 00278634	2000
*Xerotrema quercicola* Coppins and Aptroot	E 00817833	2002
*Xerotrema quercicola*	E 00817832	2002
*Xerotrema quercicola* (HOLOTYPE)	E 00278636	2006

**Table 2:** Results of Illumina sequencing showing the taxonomic name assigned to the sample, the top BLASTn hit, type of DNA extraction, Qbit score, length of ITS2 sequences, number of sequences generated per samples, number and percent of sequences matching the target species, whether Sanger sequences of the entire ITS or ITS2 were generated, and GenBank accession number.

Taxonomic Name	Top BLASTn Hit	DNA Extraction	Qubit (ng/µL)	Length	Sequences per Sample	Sequences of Target (%)	Sanger Sequences	GenBank Number
*Discina repanda* ^X^	inconclusive	NaOH + EZNA	1.65	329–355	9050	0	no	N/A
*Gyromitra arctica*^X^ (HOLOTYPE)	*G. infula*	EZNA	3.08	329	21406	20821 (97%)	yes (ITS2)	OP265173
*Gyromitra korshinskii*^X^ (HOLOTYPE)	*G. sphaerospora*	EZNA	too low	362–363	16467	14400 (87%)	yes (ITS)	OP265174
*Gyromitra leucoxantha* (HOLOTYPE)	*G. leucoxantha*	NaOH *	too low	339	12780	5934 (46%)	no	OP265175
*Gyromitra perlata* (NEOTYPE)	no PCR amplification	NaOH *	6.56	N/A	N/A	N/A	no	N/A
*Gyromitra ussuriensis*^X^ (NEOTYPE)	*G. gigas*	EZNA	2.02	349	11830	2769 (23%)	no	ON527922
*Gyromitra ussuriensis* ^X^	*G. perlata*	EZNA	too low	337	12784	6699 (52%)	no	OP265176
*Lasiosphaeria lanuginosa* (+control)	*L. lanuginosa*	EZNA	too low	288	11826	11814 (99%)	yes (ITS)	OP265177
*Propolis* sp.	inconclusive	EZNA *	too low	N/A	13513	0	no	N/A
*Stictis cylindrocarpa* ^X^	inconclusive	EZNA *	too low	N/A	12985	0	no	N/A
*Stictis fulva*^X^ (ISOTYPE)	inconclusive	EZNA *	too low	N/A	17124	0	no	N/A
*Stictis fulva* ^X^	inconclusive	EZNA *	too low	N/A	16022	0	no	N/A
*Xerotrema megalospora* ^X^	inconclusive	EZNA *	too low	N/A	6716	0	no	N/A
*Xerotrema megalospora* ^X^	inconclusive	EZNA *	too low	N/A	14458	0	no	N/A
*Xerotrema megalospora* ^X^	inconclusive	EZNA *	too low	N/A	17815	0	no	N/A
*Xerotrema megalospora* ^X^	inconclusive	EZNA *	too low	N/A	20123	0	no	N/A
*Xerotrema megalospora* ^X^	inconclusive	EZNA *	too low	N/A	32862	0	no	N/A
*Xerotrema quercicola* ^X^	inconclusive	EZNA *	too low	N/A	13786	0	no	N/A
*Xerotrema quercicola* ^X^	inconclusive	EZNA *	too low	N/A	35694	0	no	N/A
*Xerotrema quercicola*^X^ (HOLOTYPE)	inconclusive	EZNA *	too low	N/A	15442	0	no	N/A

^X^ Sequences do not exist in GenBank for these taxa. * Zymo Clean & Concentrator kit used after DNA extraction.

## Data Availability

The Sanger sequence data generated in this study are openly available in NCBI at: https://www.ncbi.nlm.nih.gov/. Raw Illumina reads have been deposited into the NCBI Sequence Read Archive (SRA) database (BioProject ID: PRJNA870474), and can be accessed via the following: http://www.ncbi.nlm.nih.gov/bioproject/870474.
